# Low coverage of *Janani Suraksha Yojana* among mothers in 24-Parganas (South) of West Bengal in 2009

**DOI:** 10.1186/1753-6561-6-S1-O3

**Published:** 2012-01-16

**Authors:** Dilip K Mandal, Prabhdeep Kaur, Manoj V Murhekar

**Affiliations:** 1 Department of Health and Family Welfare, Government of West Bengal, Bengal, Kolkata, India; 2 ICMR School of Public Health, Chennai, India; 3 ICMR School of Public Health, Chennai, India

## Introduction

*Janani Suraksha Yojana* (JSY), a maternity protection scheme, aims to reduce maternal and infant mortality and to promote institutional delivery in India. It was started in 2005. Under JSY, pregnant mothers of poor, scheduled caste and tribes shall get financial benefits of INR 500 (USD 10.7) after completing three antenatal care visits; INR 150 (USD 3.2) to INR 350 (USD 6.4) for transport to government or accredited private healthcare institution; and INR 500 (USD 10.7) for opting institutional delivery.

We evaluated JSY in South 24-Parganas district of West Bengal.

## Methods

We sampled 256 mothers who were eligible to receive JSY benefits and who had a child below one year of age. We used cluster sampling using population proportionate to size method to select these mothers. In total, 32 clusters with a cluster size of eight were identified. Sample size was calculated to achieve 95% confidence interval and 80% power, using 70% utilisation of JSY, rate of homogeneity (high utilisation of health care service) as 0.3 and design effect as 3.1. In each cluster, we selected mothers randomly. Trained personnel interviewed mothers using semi-structured questionnaire.

For stakeholder analysis, we randomly selected 97 female health worker (auxiliary nurse midwives), 32 block medical officers and public health nurses, 12 *gram panchayat pradhans* (elected representatives in local governments) and district nodal officers for JSY. We interviewed these stakeholders using a semi-structured questionnaire.

We also analysed performance of JSY and reproductive and child health in the district using secondary data from the records, registers and reports at different levels. We analysed trends in institutional delivery and coverage of JSY.

## Results

We found that 57% (147) of 256 mothers who were eligible to receive JSY benefits were registered with health services within 12 weeks of pregnancy; 85.9% (220) mothers completed at least 3 antenatal care visits; and 96.9% (248) mothers had completed one dose of tetanus toxoid injection. Blood pressure was recorded in 72% mothers; weight and haemoglobin was measured for 88% and 42% mothers respectively.

We found that 78% (202) of 256 mothers were registered under JSY and 73% (188) got JSY benefits after three antenatal care visits. Of mothers (188) who received JSY benefits, 11% (20) got financial benefits during antenatal period. Of 49% (99) mothers who delivered in healthcare institutions, 51% (51) got financial benefits for referral transport as well as institutional delivery after childbirth; 29 mothers received it before the discharge from institution. Institutional delivery was low among Muslims women (OR- 0.41, 95% CI: 0.23-0.72). Of 256 mothers 50% (128) had home delivery.

Regarding awareness on JSY, we found that 90% (231) mothers knew that some financial benefits are being given to pregnant mothers but only 64% (164) mothers had heard the name of JSY. Husband's education (Odds Ratio-2.5, 95%CI: 1.2-5.1) and knowledge of JSY (OR-11.2, 95%CI: 5.9-21.9) was positively associated likelihood of mothers getting JSY benefits.

We found that all female health workers, 94% of block medical officers and block public health nurses, and 58% of *gram panchayat pradhan* had correct knowledge of JSY. District nodal officer, 55% (53) female health workers and 47% (15) of block officials experienced inadequate and late allotment of fund for JSY. Most (88%) female health workers identified non-availability of necessary documents as a barrier to receipt of financial benefits from hospital. Disbursement of financial benefits of JSY was made on daily basis from 62% (23/37) of healthcare institutions. District or state nodal officer did not crosscheck any beneficiary.

We found that as per secondary data number of mothers receiving JSY benefits increased from 1959 in June 2007 to 14280 in September 2009. We also noted an increase in institutional delivery from 29% in April 2008 to 36% in October 2009. However maternal mortality ratio was 108 per 100000 live births during January 2007 and October 2009. We found reduction in reporting of Infant death from 2167 in 2007-2008 to 1604 in 2008-2009.

## Discussion

We find that inadequacy of fund for JSY and delayed payments of financial benefits lead to low coverage of JSY. Institutional delivery increased with decrease in infant mortality after implementation of JSY. We recommend adequate fund allotment, timely disbursement of financial benefits to beneficiaries, circulation of JSY guidelines and reorientation of stakeholders.

